# Drug-resistance in Streptococcus pneumoniae isolates among Spanish middle aged and older adults with community-acquired pneumonia

**DOI:** 10.1186/1471-2334-9-36

**Published:** 2009-03-25

**Authors:** Angel Vila-Corcoles, Ferran Bejarano-Romero, Elisabeth Salsench, Olga Ochoa-Gondar, Cinta de Diego, Frederic Gomez-Bertomeu, Xavier Raga-Luria, Xavier Cliville-Guasch, Victoria Arija

**Affiliations:** 1 Research Unit, Primary Health Care Service of Tarragona-Valls, Institut Catalá de la Salut, Tarragona, Spain; 2 Pharmacy Unit, Primary Care Department of Camp de Tarragona, Institut Català de la Salut, Tarragona, Spain; 3 Department of Laboratory and Microbiology, Hospital Joan XXIII. Tarragona, Spain; 4 Department of Laboratory and Microbiology, Hospital Santa Tecla. Tarragona, Spain; 5 Preventive Medicine and Public Health Unit. Medicine Faculty. Rovira i Virgili University, Tarragona, Spain

## Abstract

**Background:**

Pneumococcal diseases remain a major cause of morbidity and mortality worldwide. Updated data on drug-resistance from different populations may be important to recognize changes in disease patterns. This study assessed current levels of penicilin resistance among *Streptococcus Pneumoniae *causing pneumonia in Spanish middle age and older adults.

**Methods:**

Antimicrobial susceptibility was tested for 104 consecutive isolates of Streptococcus pneumoniae recovered from patients 50 years or older with radiographically confirmed pneumonia in the region of Tarragona (Spain) between 2002 and 2007. According to the minimum inhibitory concentration of tested antimicrobials (penicillin, erythromycin, cefotaxime and levofloxacin) strains were classified as susceptible or resistant. Antimicrobial resistance was determined for early cases (2002–2004) and contemporary cases (2005–2007).

**Results:**

Twenty-seven (25.9%) were penicillin-resistant strains (19 strains with intermediate resistance and 8 strains with high resistance). Penicillin-resistance was higher in 2002–2004 than in 2005–2007 (39.5% vs 18.2%, p = 0.017).

Of 27 penicillin-resistant strains, 10 (37%) were resistant to erythromycin, 8 (29.6%) to cefotaxime, 2 (7.4%) to levofloxacin, and 4 (14.8%) were identified as multidrug resistant. Case-fatality rate was higher among those patients who had an infection caused by any penicillin susceptible strain (16.9%) than in those with infections due to penicillin-resistant strains.

**Conclusion:**

Resistance to penicillin among Streptococcus pneumoniae remains high, but such resistance does not result in increased mortality in patients with pneumococcal pneumonia.

## Background

*Streptococcus pneumoniae *is the most frequent isolate from clinical samples of respiratory tract infection, including acute exacerbations of chronic bronchitis and community-acquired pneumonia [1-3]. The emergence of multiple drug-resistance has complicated the empirical treatment of pneumococcal infections. Several surveillance programs that span numerous countries indicate that the proportion of drug-resistant *Streptococcus pneumoniae *isolates continues to increase worldwide[4]. However, in Spain, where there was a great increase of penicillin-resistance in 1980s, stabilization in 1990s and a decrease in penicillin-resistant isolates in the last few years has been reported [5-7].

In elderly people, the population group with highest rates of pneumococcal infection, the penicillin intermediate-susceptibility strains has not been associated with increased mortality or unsuccessful treatments, so these strains can be treated with β-lactams. If we consider penicillin-resistant pneumococci, data reported on mortality has not been uniform[4,8,9].

Updated data about drug-resistance in *Streptococcus pneumoniae *isolates from different populations may be important in recognizing changes in disease patterns. The present study describes current levels of drug resistance among *Streptococcus pneumoniae *causing community-acquired pneumonia in Spanish middle aged and older patients, and evaluates clinical factors associated with increasing drug resistance or mortality.

## Methods

Hospital case-series that included 104 consecutively recruited isolates of *Streptococcus pneumoniae *recovered from patients 50 years or older with a radiographycally confirmed community-acquired pneumonia in the region of Tarragona (a mixed residential industrial urban area in the Northeast Mediterranean coast of Spain) with Antimicrobial susceptibility to penicillin tested between January 2002 and April 2007.

The isolates of *S. pneumoniae *were identified from blood and sputum samples in the microbiology laboratory of two reference hospitals in the study area (Joan XXIII and Sant Pau i Santa Tecla) by standard methods. Pneumococcal serotyping was carried out by the Quellung reaction using the Statens Serum Institute (Copenhagen, Denmark) typing sera. Penicillin susceptibility was determined by the Kirby-Bauer disk diffusion method with a disk containing 1 μg oxacillin (BD BBL, Sparks, MD USA). Isolates were defined as resistant when the growth inhibition diameter was 19 mm or less. Minimal inhibitory concentration (MIC) for penicillin and cefotaxime was determined by E-test method (AB Biodisk, Solna, Sweden) following the manufacturer's instructions. Penicillin-susceptible was considered when MIC ≤ 0.06 μg/mL, penicillin intermediate-resistant when MIC 0.12 – 1 μg/mL and high resistance when MIC ≥ 2 μg/mL. Cefotaxime susceptibility was considered when MIC ≤ 1 μg/mL, cefotaxime intermediate when MIC 2 μg/mL and cefotaxime resistant when MIC ≥ 4 μg/mL. Erythromycin and levofloxacin susceptibility was determined by the Kirby-Bauer disk diffusion method with a disk containing 15 μg erythromycin and 5 μg levofloxacin respectively (BD BBL, Sparks, MD USA). Erythromycin susceptibility was considered when the growth inhibition diameter was ≥ 21 mm, erythromycin intermediate between 16 and 20 mm, and erythromycin resistant ≤ 15 mm. Levofloxacin susceptibility was considered when the growth inhibition diameter was ≥ 17 mm, levofloxacin intermediate between 14 and 16 mm, and levofloxacin resistant ≤ 13 mm, according to the guidelines established by the National Committee for Clinical and Laboratory Standards (formerly NCCLS)[10]. Multidrug resistance was defined as intermediate resistance or resistance to penicillin plus intermediate resistance or resistance to ≥ 2 antimicrobial agents[11].

Cases were validated by checking clinical records and, according to the presence of underlying medical conditions, all patients were grouped into 3 risk strata on the basis of the degree of immunocompromise and risk for pneumococcal disease. High-risk level included persons with conditions associated with possible immunocompromise: immunodeficiency (including AIDS), asplenia, cancer (solid organ or haematological neoplasia), chronic nephropathy (nephrotic syndrome, renal failure, dialysis or transplantation), and long-term corticosteroid therapy (20 mg/day of prednisone or equivalent). Moderate-risk level included patients without a level 1 condition but who had a history of chronic lung disease (chronic bronchitis, emphysema or asthma), liver disease (cirrhosis or alcoholic hepatitis), heart disease (congestive heart failure or chronic angina) and diabetes mellitus. Low-risk level included patients without the above mentioned risk conditions.

Pneumonia severity index (PSI) was calculated according to criteria described in classical meta-analysis, and case-fatality was considered when the patient died (in-hospital or not) within the first 30 days after the diagnosis of pneumonia[12,13].

Penicillin-resistance was determined for early cases (2002–2004) and contemporary cases (2005–2007). For the statistical analysis, continuous variables were compared by Student's t test, whereas categorical values were compared using chi-squared or Fisher's test as appropriate.

The study was approved by the ethical committee of the Catalan Health Institute and was conducted in accordance with the general principles for observational studies set out by this institution (Exp FIS 050231).

## Results

### Patient characteristics

Median age of case patients was 71.9 years (SD: 11.6), and 75 (72.1%) were male. Fifty-nine (56.7%) were 50–74 years-old, and 45 (43.3%) were 75 years or older. Five cases were managed as outpatient and 99 were hospitalised (20 of which required admission to the ICU). Overall case-fatality rate was 15.4% (40% among those admitted in the ICU).

The most prevalent underlying conditions were chronic pulmonary disease (33.7%), smoking (28.8%), chronic hearth disease (25%) and diabetes mellitus (21.2%). Thirty-one cases (29.8%) were assigned to high-risk level, 46 (44.2%) to moderate-risk level and 27 (26%) to low-risk level. Twenty-five (24.1%) patients were assigned to PSI classes' I-III and 79 (75.9%) to classes IV and V (table 1).

**Table 1:** Clinical characteristics of patients with pneumococcal pneumonia according to penicillin susceptibility.

	Penicillin-Susceptible strains N = 77	Penicillin-Resistant strains N = 27	P-value*	Overall Strains N = 104
**Age (yrs), mean (SD)**	70.3 (11.8)	76.4 (9.5)	0.018	71.9 (11.6)

**Sex:**				
**Male**	56 (72.7)	19 (70.4)	0.814	75 (72.1)
**Female**	21 (27.3)	8 (29.6)		29 (27.9)

**Underlying conditions**				
Chronic pulmonary disease	26 (33.8)	9 (33.3)	0.967	35 (33.7)
Chronic hearth disease	18 (23.4)	8 (29.6)	0.519	26 (25)
Diabetes Mellitus	15 (19.5)	7 (25.9)	0.480	22 (21.2)
Stroke	5 (6.5)	2 (7.4)	0.870	7 (6.7)
Chronic liver disease	7 (9.1)	2 (7.4)	0.949	9 (8.7)
Chronic renal disease	10 (13.0)	7 (25.9)	0.118	17 (16.3)
Cancer	8 (10.4)	4 (14.8)	0.536	12 (11.5)
Alcoholism	11 (14.3)	0 (0)	0.038	11 (10.6)
Smoker	25 (32.5)	5 (18.5)	0.169	30 (28.8)
Corticosteroid therapy	3 (3.9)	3 (11.1)	0.166	6 (5.8)

**Risk strata of patients**				
High	21 (27.2)	10 (37)	0.477	31 (29.8)
Moderate	36 (46.8)	10 (37)	0.516	46 (44.2)
Low	20 (26)	7 (25.9)	0.802	27 (26.0)
**PSI classes**				

PSI I-III	18 (23.4)	7 (25.9)	0.790	25 (24.1)
PSI IV-V	59 (76.6)	20 (74.1)		79 (75.9)

NOTE: Data are numbers (percentage) of subjects, unless otherwise indicated. SD, standard deviation. PSI, pneumonia severity index.

* P-values were calculated by using Student's t test for continuous variables and chi-squared or Fisher's test for categorical variables, as appropriate.

### S. Pneumoniae serotypes

Pneumococcal serotype was identified in 60 cases, whereas 14 cases were due to nonserotyped organisms and 30 were not serotyped. Of the 60 serotyped cases, 12 (20%) were due to type 3, seven (11.7%) due to type 1, four (6.7%) due to type 14, four (6.7%) due to type 6A, four (6.7%) due to type 8, three (5%) due to type 6B, three (5%) due to type 12F, two (3.3%) due to type 5, two (3.3%) due to serotype 4, two (3.3%) due to type 19F, two (3.3%) due to type 23A, two (3.3%) due to type 35B, two (3.3%) due to type 7F, two (3.3%) due to type 19A, two (3.3%) due to type 23B and one due to types 9V, 9N, 15B, 16, 23F, 33 and 38.

### Antimicrobial susceptibility

Of the total 104 cases, 27 (25.9%) were penicillin-resistant strains: 19 strains with intermediate resistance and 8 strains with high resistance (four cases with MIC = 2 μg/mL; four cases with MIC = 4 μg/mL)

Of the 100 isolates tested for erythromycin, 19 (19%) were resistant. For levofloxacin, 89 isolates were tested and five (5.6%) were resistant. Among the 93 isolates tested for cefotaxime, nine (9.7%) were resistant (table 2).

**Table 2:** Antimicrobial resistance to cefotaxime, erythromycin and levofloxacin observed among pneumococcal strains isolated over the study period.

	All isolates (N = 104)		Penicillin resistant isolates (N = 27)	
	
	Tested	Resistant strains	Tested	Resistant strains
**Cefotaxime**	93	9 (9.7)	27	8 (29.6)

**Erythromycin**	100	19 (19)	27	10 (37)

**Levofloxacin**	89	5 (5.6)	24	2 (8.3)

NOTE: Data are numbers (percentage) of strains.

Only four (14.8%) isolates were identified as multidrug resistant *S. pneumoniae *(MRSP). Of these, one case was highly resistant to penicillin, cefotaxime and levofloxacin and three cases were highly resistant to penicillin, erythromycin and cefotaxime. Median age of patients with MRSP was 76.8 years, all of them had an underlying disease (one was current smoker, one had chronic heart disease and two had a chronic pulmonary disease), three were non-bacteremic cases and only one died. In three cases the microorganism was resistant to the initial antimicrobial regimen, and one of them died. Only two MRSP were serotyped (6B both of them).

Globally, 76 specimens were recovered from blood samples and 28 from sputum samples. Of the 42 resistant strains at least to one antibiotic, 26 (62%) were isolated from blood and 16 (38.1%) were from sputum. Although the difference did not reach statistical significance, penicillin resistance was substantially higher among *Streptococcus pneumoniae *recovered from sputum than from blood (44.4% *vs. *24.2%, p = 0.186).

Comparing penicillin-susceptible and non-susceptible strains, statistically significant differences were observed only for age (table 1). Of the 19 serotyped strains with penicillin resistance, four (21.1%) were serotype 14, three (15.8%) were serotype 23B, three (15.8%) were serotype 6B, two (10.5%) were serotype 19F, two (10.5%) were serotype 6A, and one were serotypes 1, 3, 8, 9V and 35B.

Penicillin-resistance was substantially lower in contemporary isolates than in early isolates (18.2% vs 39.5%, p = 0.017). Of the 38 early isolates, 10 strains with intermediate resistance and five strains with high resistance. Of the 66 contemporary strains, 9 strains with intermediate resistance and three strains with high resistance. Among patients 50–74 years old and among patients with moderate risk this decrease was statistically significant (table 3).

**Table 3:** Comparison of patients with pneumococcal pneumonia caused by penicillin-resistant strains in early and contemporary isolates according to their age and risk stratum.

	Early isolates (2002–2004)		Contemporary isolates (2005–2007)		
		
Strata	Overall strains	Penicillin-resistant strains	Overall strains	Penicillin-resistant strains	P-value*
**Age**					
50–74 yrs	17	7(41.2)	42	2 (4.8)	0.001
75 yrs or more	21	8 (38.1)	24	10 (41.7)	0.807

**Risk strata**					
High	11	4 (36.4)	20	6 (30)	0.717
Moderate	20	8 (40)	26	2 (7.7)	0.008
Low	7	3 (42.9)	20	4 (20)	0.235

**Overall**	38	15 (39.5)	66	12 (18.2)	0.017

NOTE: Data are numbers (percentage) of subjects.

* P-values were calculated by using Student's t test for continuous variables and chi-squared or Fisher's test for categorical variables, as appropriate.

### Impact of resistance on severity outcomes

Mean length of hospital stay was slightly higher among patients infected with penicillin-resistant strains than in penicillin-susceptible strains (16.9 vs. 13.5 days; p = 0.358). PSI score did not differ in both groups (112.5 *vs. *111.1; p = 0.838).

Overall mortality was 15.4%. One patient died among the 8 cases with an inadequate initial antimicrobial therapy, based on antibiogram results. Although it was not statistically significant, mortality was considerably higher among those patients who had an infection caused by a penicillin susceptible strain than in those with infections due to penicillin-resistant strains (16.9% *vs. *11.1%; p = 0.474).

### Antimicrobial therapy

The antimicrobial therapies initially used in the 104 patients were: levofloxacin in 26 cases (25%), an association of macrolide and cephalosporin in 18 cases (17.3%), amoxicillin clavulanic acid in 14 cases (13.5%), a cephalosporin in 14 cases (13.5%), an association of macrolide and amoxicillin clavulanic acid in 14 cases (13.5%) and other treatments or associations in 18 cases (17.3%).

Considering the 8 cases classified as high-resistance to penicillin, antimicrobial therapy initially used after CAP diagnosis was levofloxacin in 3 cases, amoxicillin clavulanic acid in two cases, an association of macrolide and cephalosporin in two cases, and one case was treated with tazobactam. One patient (a man 88 years-old, PSI class V, treated with amoxicillin clavulanic acid, with bacteremic pneumococcal pneumonia due to serotype 6B, resistant to penicillin (MIC = 2) and resistant to cefotaxime, erythromycin and clindamycin) died two days after hospital admission, whereas the remaining 7 patients were non-fatality cases.

Considering the 19 cases classified as intermediate-resistance to penicillin, antimicrobial therapy initially used after CAP diagnosis was cephalosporin in 5 cases, amoxicillin clavulanic acid in 4 cases, an association of macrolide and cephalosporin in 4 cases, levofloxacin in 2 cases, and other treatments or associations in 4 cases. Two of these patients, both treated with a third generation cephalosporin, died within 10 days after hospital admission (one case was a man 67 years old with bacteremic pneumonia, PSI class V, due to serotype 14; and an other case was a man 76 years-old with bacteremic pneumonia, PSI class IV, due to serotype 6A), whereas the remaining 17 penicillin intermediate resistance cases were non-fatality cases.

## Discussion

In the present study, the overall rate of penicillin-resistance among isolates of *Streptococcus pneumoniae *was considerable (25.9%). The proportion of penicillin-resistance was higher among early isolates than in contemporary isolates, which concurs with other Spanish studies[5,7,14]. Among Spanish individuals, Fenoll et al[7] found that a high-level penicillin-resistance rate (MIC > 2 mg/l) decreased from 13.3% in 1997 to 12.9% in 2001. In Catalonia, the high-level penicillin-resistance fell from 15% (1989–1993)[3] to 8.9% (1999–2002)[14], which fits with the 9% found in our study. In contrast, the results found in other countries showed an increase in penicillin-resistance rate. In United States, the high-level penicillin-resistance increased from 14.7% (1998–1999) to 18.4% (2001–2002)[15]. Although several factors explain the emergence of drug resistant *Streptococcus pneumoniae*, the single most important consideration, however, is the selective pressure of use of antimicrobial agents. Reductions in the rate of resistance found in our study could be explain by a reduction in consumption of antibiotics in Catalonia, going from 17.77 defined daily doses per 1000 inhabitants per day (DHD) in 1999 to 12.58 DHD in 2006, and could be partially attributed to the vaccination that covers serotypes typically associated with drug resistance[14].

We found a statistically higher age among patients infected with penicillin-resistant strains than in those infected with penicillin-susceptible strains. However, because of low statistical power, we could not reject that prevalence of underlying conditions was the predisposing cause for this age-related differences in the aetiology of pneumococcal pneumonia.

In accordance with previous studies[5,14] we found a high proportion of underlying diseases among patients with pneumococcal pneumonia. Although not statistically significant, the proportion of penicillin resistance in our study was higher among patients with low or moderate level risk in early isolates. Conversely, in contemporary isolates this proportion was higher among patients in high level of risk. Some studies[6,16] have reported that patients with chronic pulmonary disease, HIV infected, suspected aspiration or who were admitted to the hospital within the previous 3 months were more likely than other patients to have penicillin resistant pneumococcal pneumonia. In our study, only two patients had been hospitalized in prior 3 months, and none of them were HIV infected. Chronic pulmonary disease was highly prevalent in our study but differences were not observed in prevalence by penicillin resistance status.

Almost three quarter (74.1%) of penicillin-resistant pneumococcal pneumonia were PSI classes IV-V. There was a similar percentage of penicillin-resistant pneumonia between PSI classes IV-V and PSI classes I-III. This results agree with those found in other studies[6,16] that only reported a trend for higher PSI classes to be associated with penicillin-resistant pneumococcal pneumonia.

In the present study, the rate of penicillin susceptibility was higher in bacteremic than non-bacteremic cases (65.8% *vs. *42.9%). These fits with Aspa's report[6], where penicillin susceptibility was 71% among blood culture isolates and 50% among sputum isolates. In the USA, a higher prevalence of resistance to penicillin among lower respiratory tract than among isolates recovered from blood samples (16.2% *vs. *13.7%) has also been reported[15].

In our study, most penicillin resistant *S. pneumoniae *isolates were resistant to at least one additional antimicrobial agent, and 3.8% (four cases) of all isolates were identified as MRSP. As described in previous studies penicillin resistance, particularly high-level resistance, predicts resistance to other antimicrobials[11,14,17-19]. We found that all MRSP were highly resistant to penicillin and cefotaxime, and three of them were highly resistant to erythromycin too.

We found that an inadequate initial antimicrobial treatment was started in 7.7% of patients. Although in our study there was no excess mortality due to initial inappropriate treatment (12.5%), likely due to a small sample size, it is important to take into account underlying condition or regional pattern of drug resistance for *S. pneumoniae *in determining adequate antimicrobial therapy[11,17,20].

In accordance with other studies[5,14,21,22], penicillin resistance was not related with higher mortality. It has been suggested that the primary determinant of virulence might be the serotype rather than its sensitivity to penicillin, and it is possible that the mechanisms conferring penicillin resistance are related to those leading to a loss of virulence. Previous studies of pneumococcal bacteremia did not show differences in mortality between those with susceptible and those with non-susceptible pneumococci after adjustment for age, underlying disease, severity of illness on presentation, and appropriate concordant treatment[5,6,16].

We found that three serotypes (14, 6B and 23B) were associated with increasing risk of penicillin resistance, accounting for a 53% of serotyped pneumococci causing penicillin-resistant infections and the serotype 6B was responsible for 50% of cases of MRSP. In accordance with our results, Fenoll et al[7] reported that 56% of penicillin-resistant pneumococcal strains belonged to serotypes 6 (18%), 14 (19%) and 19 (19%). In a long term follow-up between 1983 and 2003 Mufson et al[23] found that all blood isolates of penicillin-resistant were capsular serotypes 6, 9, 14, 19 and 23.

Main limitation in this study includes the relatively low number of cases and the difficulty to generalize results. According to classical breakpoint criteria, it could be concluded that, in the study area, resistance to penicillin among Streptococcus pneumoniae remains high, although such resistance does not result in increased severity or mortality. However, it must be emphasized that, according to the new breakpoint criteria for intravenous penicillin[24], almost all strains in our study would be susceptible.

To date, criteria for penicillin susceptibility of Streptococcus pneumoniae remains unchanged for patients without meningitis who can be treated with oral penicillin (e.g., for outpatient pneumonia). However, since 2008, for patients without meningitis who are treated with intravenous penicillin, the new breakpoints are ≤ 2, 4, and ≥ 8 μg/mL for susceptibility, intermediate-resistance and high-resistance, respectively[24]. Thus, considering the new pneumonia breakpoints, 100 (96.2%) of the 104 cases in our study would be penicillin-susceptible (MIC ≤ 2 μg/mL), 4 (3.8%) would be penicillin intermediate-resistant (MIC = 4 μg/mL) and none would be high resistance (MIC ≥ 8 μg/mL).

According to the change in pneumonia breakpoint for susceptibility[24], percentages of strains resistant to penicillin have dramatically decreased worlwide. In Spain, a large surveillance study that included 2,721 isolates of S. Pneumoniae obtained from November 2001 to October 2002 in diferent Spanish Hospitals[25] reported that (taking into account the old pneumonia breakpoint) 20% of overall strains were penicillin resistant (MIC >2 mg/mL) and 24% were intermediate resistant strains (MIC = 0.12–1 mg/mL). However, if we consider the new pneumonia breakpoint, 94.3% of the total strains in the forementioned study would be currently considered penicillin susceptible, which is also in agreement with data found in the present study (96.2% penicillin susceptible strains in our study considering the new breakpoint).

## Conclusion

According to classical pneumonia breakpoints for susceptibility to penicillin, the resistance among Streptococcus pneumoniae was considerable, but with the use of new breakpoints, the percentage of Streptococcus pneumoniae penicillin-resistant decreases dramatically.

In vitro penicillin resistance is not associated with increasing mortality, even considering new breakpoints for susceptibility to intravenous penicillin.

The low mortality rates reported among patients with pneumonia due to penicillin resistant strains could likely be due to the fact that patients were treated with active agents, such as penicillins or third generation cephalosporins.

## Competing interests

The authors declare that they have no competing interests.

## Authors' contributions

AV-C, FB, ES, OO-G and CdD designed the study, assessed outcomes, and wrote and edited the paper. FG-B, XR and XC obtained the data. VA did the statistical analysis. AV-C coordinated the study.

## Pre-publication history

The pre-publication history for this paper can be accessed here:

http://www.biomedcentral.com/1471-2334/9/36/prepub
